# Capillaria hepatica (Calodium hepaticum) infection in a horse: a case report

**DOI:** 10.1186/s12917-017-1301-3

**Published:** 2017-12-08

**Authors:** Akihiro Ochi, Tatsuro Hifumi, Takanori Ueno, Yoshinari Katayama

**Affiliations:** 1 Equine Research Institute, Japan Racing Association, 1400-4 Shiba, Shimotsuke, Tochigi, 329-0412 Japan; 2 Laboratory of Veterinary Histopathology, Joint Faculty of Veterinary Medicine, Kagoshima University, Kagoshima, 890-0065 Japan

**Keywords:** *Capillaria hepatica*, Hepatic capillariasis, Horse

## Abstract

**Background:**

*Capillaria hepatica* is a zoonotic parasite in humans and animals and has a worldwide distribution. However, infections in mammals apart from rodents, which are natural hosts of the parasite, have rarely been reported. This report describes the first known case of *C. hepatica* infection in a horse in Japan.

**Case presentation:**

A 3-year-old filly without clinical signs was presented at a slaughterhouse in Japan. Gross examination revealed white to tan nodules 0.5 to 1.5 cm in diameter in the parenchyma of the liver. Histologically, the nodules had mature fibrous capsules and consisted of multifocal to coalescing granulomatous inflammations with numerous nematode eggs. The eggs were barrel shaped with an opercular plug on each end and double-layered shells; these findings are consistent with the features of *C. hepatica* eggs.

**Conclusions:**

To our knowledge, this is the first case of *C. hepatica* infection in a horse in Japan. The pathological findings confirmed the presence of this pathogen in this part of the world, and they highlight the importance of this nematode in the differential diagnosis of hepatic granulomatous lesions in horses.

## Background

*Capillaria hepatica* (now called *Calodium hepaticum*) is a nematode in the family Capillariidae and a zoonotic parasite with a worldwide distribution. The usual hosts for the parasite are rodents, especially wild rats and mice [1], but the parasites uncommonly infect various other mammals including humans [2]. In a review of *C. hepatica* infections in humans, 163 cases, including 72 genuine infections have been reported in Europe, North and South America, Asia and Oceania, especially in tropical and temperate zone [1]. In veterinary medicine, this parasite was found in at least 69 species in 25 mammalian families including Insectivora, Chiroptera, Lagomorpha, Artiodactyla, Perissodactyla, Hyracoidea, Marsupialia, Carnivora, and Primates [2]. The life cycle of *C. hepatica* is direct [3]. After the animal ingests embryonated eggs, larvae hatch in the intestine and migrate to the liver, where they mature, breed, and lay unembryonated eggs. These eggs are not passed in the feces of the host but remain in the liver until the animal dies and decomposes or the host is eaten by scavengers or predators. After the death of the host, the unembryonated eggs are released into the environment and become infective and embryonated. In contrast, ingestion of unembryonated eggs leads to spurious infection, and the non-infective eggs are shed into the environment with the feces.

*Capillaria hepatica* is a zoonotic nematode that inhabits the liver of the host during the adult stage of the life cycle. The presence of worms and eggs can provoke focal necrosis, fibrosis, and inflammatory reaction in the liver [4] and result in hepatic capillariasis in a variety of animals [1]. However, *C. hepatica* infection in horses is relatively rare and only two cases have been reported, in the United Kingdom and Canada [5, 6]. This case report describes the first known case of *C. hepatica* infection in a horse in Japan.

## Case presentation

The subject was a 3-year-old female Thoroughbred presented at a municipal slaughter plant in Japan. It was in good body condition, showed no signs of illness, and passed ante-mortem inspection. On gross examination, within the liver there were multifocal, firm, homogenous white to tan nodules 0.5 to 1.5 cm in diameter scattered in the parenchyma. On the cut surface, the nodules were rich in fibrous tissue with partial calcification. The nodules were harvested from the horse, fixed in 20% neutral buffered formalin, and sent to the Equine Research Institute of the Japan Racing Association for histological examination. After fixation, the nodules were decalcified in Morse’s solution. All samples were embedded in paraffin wax, cut at 4 μm, and stained with hematoxylin and eosin.

**Fig. 1:**
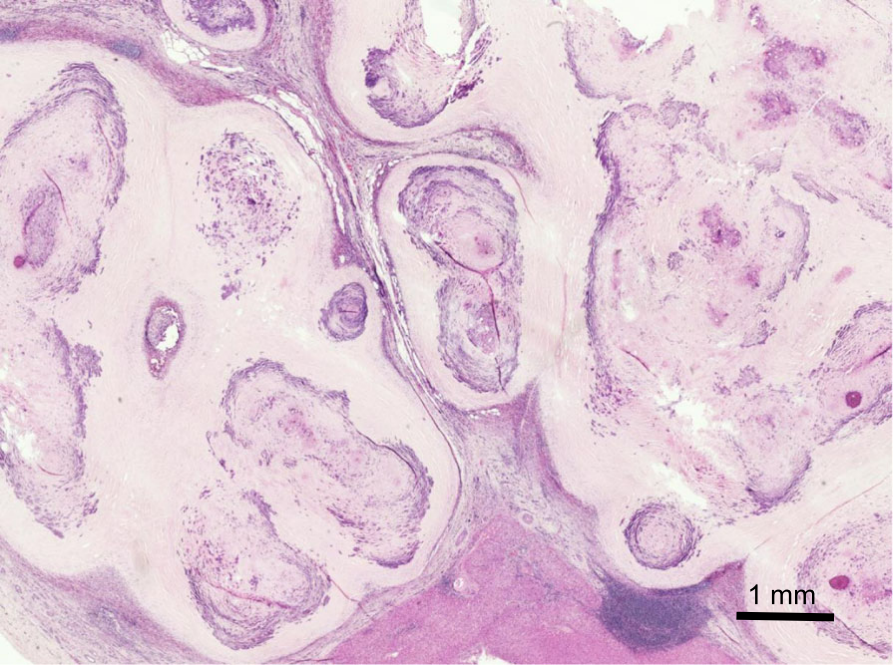
Histology of the hepatic nodules. Multifocal to coalescing granulomatous lesions, with mild calcification, are present in the hepatic parenchyma. There are infiltrations of inflammatory cells, and edema is apparent at the periphery of the nodules. Hematoxylin and eosin stain

**Fig. 2:**
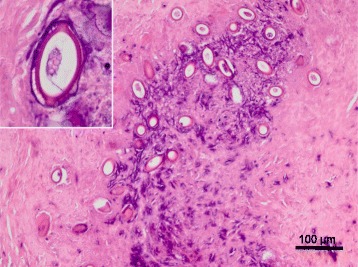
*Capillaria hepatica* eggs. Most of the eggs are surrounded by fibrous tissue. Inset: The eggs are barrel-shaped and have a thick, two-layered wall with a reticulated surface. Hematoxylin and eosin stain

Histological examination revealed multifocal to coalescing, partially calcified granulomatous lesions in the hepatic parenchyma. Each lesion measured up to about 1.5 cm and contained nematode eggs surrounded by a fibrous capsule (Fig. 1). The eggs were barrel shaped with an opercular plug at each end and a double-layered shell with a spoke-like pattern (Fig. 2); they measured about 50 by 20 μm. Lymphocytes, macrophages, and a few eosinophils were infiltrated around the lesions. Although there were no adult worms in the tissues, the eggs were identified as those of *C. hepatica* on the basis of their morphological characteristics.

## Discussion and conclusions

*Capillaria hepatica* is a parasitic nematode with a worldwide distribution [3]. Rodents are considered the main reservoirs, but the parasite rarely infects other animals. In Japan, the prevalence of *C. hepatica* in rodents is unknown, but the eggs or adult worms, or both, have been reported in mice and rats [7, 8]. Moreover, hepatic capillariasis has been observed in 2.25% of 400 cattle, although the nematode was not classified as *C. hepatica* [9]. Sekikawa et al. [10] reported the first human case of *C. hepatica* in Japan. However, to our knowledge, *C. hepatica* in horses has not been reported in this country. In horses, hepatic capillariasis is rare and only two cases have been reported—in the United Kingdom and Canada [5, 6]. The lesions consisted of fibrous connective tissue around eggs, as was seen in the present case.

Infection with *C. hepatica* occurs via the ingestion of embryonated eggs in the environment. In human cases, unsanitary practices, poor hygienic conditions, and the presence of dense rodent populations are thought to be predisposing factors of infection. [1]. In addition, the infections are usually found in children from 1 to 5 years old [11]. This higher incidence in children may be due to their more frequent soil-hand-mouth contact [12]. Children or adults with the habit of eating soil (geophagia or pica) are especially at risk of infection [13]. The habit of geophagia has been observed in horses and is considered to indicate nutritional deficiency or boredom [14]. Therefore, infection in horses is most likely due to accidental ingestion of the embryonated eggs with food, water, or soil, although the exact route in the present case was unknown.

Liver biopsy remains the gold standard method for the diagnosis of hepatic capillariasis [15], although other diagnostic methods are available in human medicine [1]. Imaging modalities such as ultrasound and computed tomography are helpful [16]. In addition, serologic methods such as indirect immunofluorescence can be used for screening purposes. However, these methods have not been widely used for diagnosis in veterinary medicine. A definitive diagnosis is commonly achieved by finding the adults or eggs, or both, in the liver.

This paper provides the first report of hepatic capillariasis in a horse in Japan. Although further investigations are needed to determine the prevalence of the nematode in horses, our findings indicate that *C. hepatica* infection should be considered in the differential diagnosis of hepatic granulomas in horses.
